# Biocompatibility Studies of Gadolinium Complexes with Iminodiacetic Acid Derivatives

**DOI:** 10.1007/s12011-018-1496-6

**Published:** 2018-09-13

**Authors:** Magdalena Markowicz-Piasecka, Agata Skupień, Elżbieta Mikiciuk-Olasik, Joanna Sikora

**Affiliations:** 1 Laboratory of Bioanalysis, Department of Pharmaceutical Chemistry, Drug Analysis and Radiopharmacy, Medical University of Lodz, ul. Muszyńskiego1, 90-151 Lodz, Poland; 2 Students Research Group, Laboratory of Bioanalysis, Department of Pharmaceutical Chemistry, Drug Analysis and Radiopharmacy, Medical University of Lodz, ul. Muszyńskiego 1, 90-151 Lodz, Poland; 3 Department of Pharmaceutical Chemistry, Drug Analysis and Radiopharmacy, Medical University of Lodz, ul. Muszyńskiego 1, 90-151 Lodz, Poland

**Keywords:** Iminodiacetic acid, Gadolinium, Biocompatibility, Magnetic resonance imaging

## Abstract

Apart from using as radiopharmaceuticals, iminodiacetic acid derivatives, after complexation with gadolinium, have been also tested as MRI CAs (magnetic resonance imaging contrast agents) since they show high affinity to hepatocytes and therefore provide high-resolution MRI of the liver. The purpose of this study was to evaluate the biocompatibility of four gadolinium complexes with iminodiacetic acid (IDA) derivatives differing in substituent in aromatic ring by estimating their influence on plasma hemostasis, integrity of erythrocyte membrane, and toxicity towards human umbilical vein endothelial cells (HUVECs). The influence of gadolinium-based CAs on plasma hemostasis was evaluated by measuring PT (prothrombin time), APTT (activated partial tromboplastin time), and TT (thrombin time). The effects of tested compounds on RBCs (Red Blood Cells) were assessed using hemolysis assay and microscopy studies. The influence of gadolinium complexes on the barrier properties of HUVECs was assessed by means of real-time method based on the measurements of the impedance changes of the cells. Gadolinium complexes did not affect significantly PT and TT. APTT measurements revealed significant prolongation in the presence of all tested gadolinium complexes at the concentration higher than 0.5 μmol/mL. Hemolysis assay showed that compounds with alkyl substituents in benzene ring without halogen atom (1–3) do not exert unfavorable effect on the integrity of erythrocyte membrane over the entire concentration range. All gadolinium complexes at 1.0 μmol/mL contribute to the decrease in HUVEC viability and integrity. To conclude, the study describes biocompatibility studies of gadolinium-based CAs, provides additional insight into their potential toxicity associated with systemic administration, and underscores the necessity for further research.

**Electronic supplementary material:**

The online version of this article (10.1007/s12011-018-1496-6) contains supplementary material, which is available to authorized users.

## Introduction

Contrast agents (CAs) are typically administered intravenously to human body which is related to instantaneous contact with blood tissue (red blood cells (RBCs), white blood cells (WBCs), platelets, and proteins). Therefore, a rapid sequence of events may occur. Many of these have not been fully elucidated and their potency to bind or interact with plasma proteins, blood cells, or endothelium may contribute to serious problems to blood functions [1, 2].

All newly synthesized drug molecules, particles for diagnostic purposes, various natural and synthetic polymers used in biomedical applications have to be tested for their biocompatibility [3], which can be defined as the capability of coexistence with living tissues or organisms without being toxic, injurious, or physiologically reactive and not causing immunological response. One of the issues of biocompatibility constitutes blood compatibility, known also as hemocompatibility [3]. Several interacting defense mechanisms or systems are activated upon the exposition of new compound to blood. According to Dawids [4], these systems have been divided into the coagulation system, the mutually complement system, the kinin-kallikrein system, and cellular systems. The ideal tested compound should not release any toxic chemicals or fragment particles into the body; induce an excessive immune, inflammatory, thrombogenic, or fibrogenic response [4]. Therefore, the development of novel hemocompatible drugs or devices is regarded as one of the most challenging problems of the contemporary pharmaceutical sciences.

Different literature sources suggest various tests to conduct for devices having direct contact with circulating blood. For instance, US Food and Drug Administration [5] recommends to carry out hemolysis, complement activation, and thrombogenicity testing. In turn, Dawids [4] and Williams [6] present the tests for activation of hemostasis (e.g., the clotting cascade, platelet adhesion, and activation of the fibrinolytic system), interactions with proteins, and in vitro test for toxicity assessment (e.g., toxicity towards erythrocytes).

It has been documented that CAs might interact with the coagulation process, lead to platelet activation and degranulation [7]. For instance, Krause et al. [8] determined the effect of 12 contrast agents (e.g., ionic monomeric and dimeric CAs, nonionic monomeric, and nonionic dimeric x-ray contrast agents, as well as MRI agents: gadolinium (Gd)-DTPA, Gd-EOB-DTPA, and gadobutrol) on Prothrombin Time (PT), bleeding time, and clot stability. The authors found that nonionic contrast agents (x-ray and MRI) increased the PT by a factor of 1.5 to 2, whereas ionic compounds prolonged PT by a factor of > 3. Prolongation of bleeding time lasted as long as 24 h for some CAs. Krause et al. [8] stated also that clots formed in the presence of iopromide were unstable and did not absorb the contrast agent [8]. It has also been reported that ionic contrast media inhibit both the intrinsic and extrinsic coagulation pathway at several level and may act as direct inhibitors of thrombin production [9]. In the case of erythrocytes, it was found that CAs may contribute to the changes in their morphology which as a consequence can change their capacity for oxygen delivery and pH buffering [10].

Iminodiacetic acid derivatives, including mebrofenin after radiolabeling with technetium (^99m^Tc), can be used as radiopharmaceuticals (^99m^Tc-bromotriethyl-IDA [mebrofenin]) during cholescintigraphy [11, 12]. The review of literature indicates that (^99m^)Tc-mebrofenin is the most valuable quantitative radiotracer for functional examination of the liver [13]. Iminodiacetic acid derivatives, after complexation with gadolinium, have been also tested as MRI CAs since they show high affinity to hepatocytes and therefore provide high-resolution MRI of the liver [14–16].

**Fig. 1:**
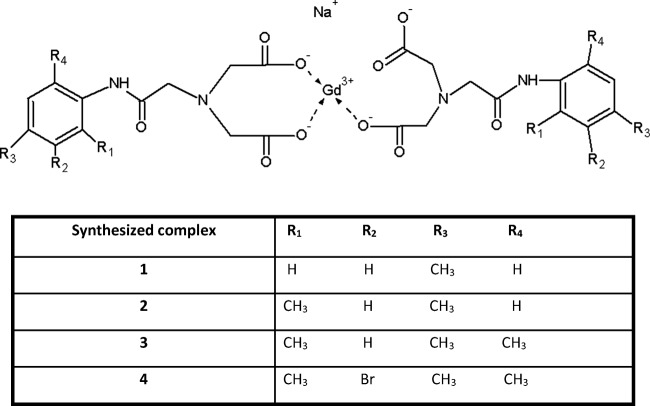
Gadolinium complexes **1**–**4**: sodium salt of N-(4-methylacetanilide)iminodiacetate gadolinium–**1**, sodium salt of N-(2,4-dimethylacetanilide)iminodiacetate gadolinium-**2**, sodium salt of N-(2,4,6-trimethylacetanilide)iminodiacetate gadolinium-**3**, sodium salt of N-(3-bromo-2,4,6-trimethylacetanilide)iminodiacetate gadolinium–**4**. The sythesized complexes were obtained in a form of hexahydrates

Within this paper, we present the biocompatibility study of four gadolinium complexes with derivatives of iminodiacetic acid (Fig. 1). The studies included evaluation of gadolinium-based CAs on the selected parameters of plasma hemostasis including prothrombin time (PT), activated partial thromboplastin time (APTT), and thrombin time (TT). Furthermore, we evaluated the influence of the compounds on stability of erythrocyte membrane and morphology of RBCs. The final part describes the effects of gadolinium complexes on the barrier properties of human umbilical vein endothelial cells (HUVECs), which may provide evidence of the toxicity of the tested CAs towards endothelium after intravenous administration.

## Materials and Methods

### Materials

For APTT assays, we used Bio-Ksel System APTTs reagent and calcium chloride (Bio-Ksel, Grudziądz, Poland). Bio-Ksel PT plus reagent (tromboplastin and solvent) was used in PT tests, while thrombin (3.0 UNIH/mL, Bio-Ksel, Poland) was utilized in TT tests. Bio-Ksel normal plasma (Bio-Ksel, Grudziądz, Poland) was used for validation of APTT, PT, and TT methods. Triton X-100 (cat. No. 841810492) was obtained from Polish Chemical Reagents (Poland).

For experiments evaluating the effects of gadolinium complexes on endothelial cells, we used: normal human umbilical vein endothelial cells (HUVEC) (Lonza, Italy), medium EGM-2-medium + bullet kit (Lonza, Clonetics, Italy), trypsin-EDTA–0.05% solution (Sigma Aldrich, Germany), trypsin neutralizing solution (Lonza, Italy), HEPES-buffered saline solution (Lonza, Italy), phosphate-buffered saline (PBS) (Bio-Med, Lublin, Poland).

### Plasma Preparation

The studies on the biological material were approved by the Bioethics Committee of the Medical University of Lodz (RNN/109/16/KE). Blood was obtained from Blood Donation Centre in Lodz. The procedure for plasma preparation for coagulology and erythrotoxicity studies was described previously [17].

### Tested Compounds

Within this study, four gadolinium complexes with iminodiacetic acid derivatives were examined (Fig. 1). The first step of synthesis involved preparation of four ligands which were as follows: N-(4-methylacetanilide)iminodiacetic acid, N-(2,4-dimethylacetanilide)-iminodiacetic acid, N-(2,4,6-trimethylacetanilide)iminodiacetic acid, and N-(3-bromo-2,4,6-trimethylacetanilide)-iminodiacetic acid [14, 16]. Gadolinium complexes were obtained using gadolinium chloride (Sigma Aldrich, Germany) as described previously [15, 16].

### Coagulation Tests: PT, INR, FBG, APTT, TT

The measurements of PT, APTT, and TT were carried out according to the routine procedure and described elsewhere [18, 19]. The tests were conducted on coagulometer (CoagChrom-3003 Bio-Ksel, Poland). In every test, control samples consisting of distilled water (solvent for gadolinium complexes) were conducted. Validation of the methods was performed using Bio-Ksel normal plasma. Coefficients of variability for the tests were estimated (PT: W = 2.78%, APTT: W = 1.08%, TT: W = 1.15%; *n* = 5). The reference values for each test according to the manufacturer are as follows: PT: 9.7–14.6 s; APTT: 26.7–40.0 s; TT: 14.0–18.0 s for 3.0 UNIH/mL of thrombin.

### Red Blood Cell Lysis Assay

RBCs were washed three times and suspended in 0.9% saline. Two percent RBC suspension was incubated at 37 °C for 1 h with the tested compounds at appropriate concentrations or 0.9% NaCl (control), following centrifugation (1000×*g*; 10 min). The amount of released hemoglobin was determined spectrophotometrically at 550 nm and percentage of hemolysis was counted. A sample of 2.0% *v*/*v* Triton X-100 was used as a positive control (representing 100% of hemolysis), whereas a sample of saline solution represented spontaneous hemolysis of RBCs (control) [14]. The coefficient of variability for the method equals: W = 9.51%, *n* = 5.

### RBC Morphology

A 2% erythrocyte suspension, prepared as for lysis assay, was incubated at 37 °C for 60 min with various concentrations of gadolinium complexes. The RBC morphology was evaluated using a phase contrast Opta-Tech inverted microscope, at 400-times magnification, equipped with software (OptaView 7) for image analysis.

### HUVEC Cell Subculturing

HUVEC cells were subcultured according to the manufacturer’s (Lonza, Italy) guideline.

### Cell Culture in the Real-Time Cell Electric Impedance Sensing System (RTCA-DP)

The effect of gadolinium complexes on endothelium monolayer was evaluated by means of electronic method conducted on RTCA DP (Real-Time Cell Analyzer (Roche & ACEA Biosciences)). RTCA DP system operates by tracking electrical impedance signals and enables the cell growth status to be evaluated in real time. The status of cell including viability, adhesion, morphology, or barrier properties is presented by Cell Index (CI), which is used to measure relative changes in electrical impedance. CI is a unitless parameter, which is counted by division of measured impedance by the value of the background [20].

The general procedure of cell culturing in RTCA DP system was previously described [21, 22]. In the first phase, HUVEC cells were seeded on E-16 plates at a density of 20,000 cells per well. HUVEC cells reached plateau phase on the second day after seeding (the value of CI reaching 7–8). Afterwards, the solutions of tested complexes dissolved in cell culturing medium were added to each well. After 48 h, the experiment was stopped and the CI was collected.

### HUVEC Morphology

The cells were seeded on 48-well plate and allowed to reach 80% confluency. Then, the medium (control) or medium containing dissolved gadolinium complexes was added. After 24 h of incubation, the cell morphology was analyzed (100-times magnification) with inverted microscope with phase contrast (Opta-Tech, software OptaView 7).

### Statistical Analysis

Statistical analysis was conducted using a commercially available package (Statistica 12.0, StatSoft, and GraphPad Prism 5). The results were expressed as the mean ± standard deviation (SD) of at least four independent experiments. The Shapiro-Wilk test was used to verify the normal distribution of continuous variables. For all variables with a normal distribution, paired *t* test and one-way ANOVA were performed. For intergroup comparison, two-way ANOVA and subsequent post hoc tests were used. The variables with non-normal distributions were compared using the Wilcoxon signed rank test. *p* value below 0.05 (*p* < 0.05) was considered statistically significant.

## Results

### Coagulation Tests (PT, APTT, TT)

**Fig. 2:**
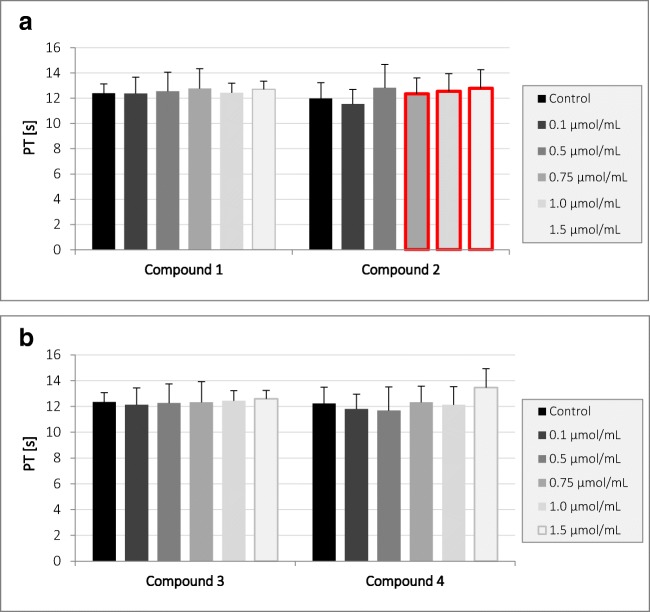
The effects of gadolinium complexes **a** compounds **1** and **2**; **b** compounds **3** and **4** on prothrombin time (PT) (mean ± SD; *n* = 5) after 3-min incubation in plasma; final volume 160 μL. Compound 2 at concentration of 0.75 μmol/mL and above significantly prolonged PT. Significant changes are marked with red frame

Studies have shown (Fig. 2) that compound **2** at a concentration of 0.75 μmol/mL and above contributes to a statistically significant prolongation of PT; however, observed significant changes of PT are enclosed within the reference values (9.7–14.6 s). Other compounds did not affect the value of PT.

Apart from PT, INR (International Normalized Ratio) was also assessed. INR is a standardized international prothrombin time index which was introduced by the international committees of ICSH (International Committee for Standardization in Hematology) and ICTH (International Conference on Transport and Health) to make the INR result independent from the applied thromboplastin and measuring methods. In our studies, the results of INR measurements confirmed results obtained for PT. In the case of the tested compounds, compound **2** displayed the statistically significant effect on the INR value. The studies revealed also that none of the compounds statistically significantly affected the level of fibrinogen in plasma (Fig. S1, Supplementary materials).

**Fig. 3:**
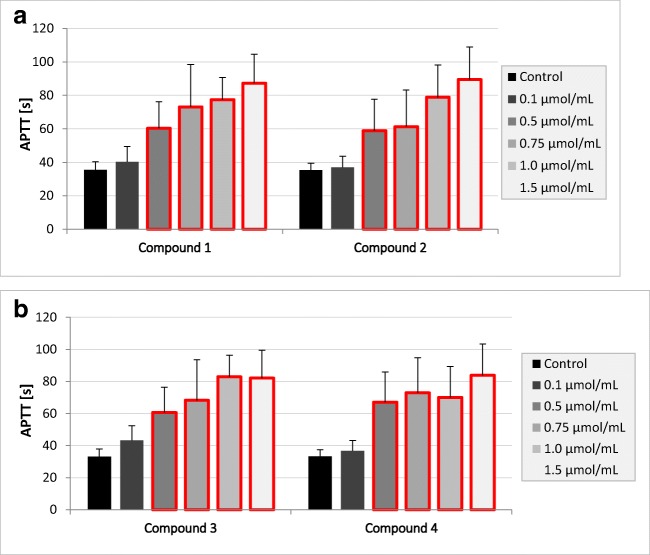
The effects of gadolinium complexes **a** compounds **1** and **2**; **b** compounds **3** and **4** on activated partial thromboplastin time (APTT) (mean ± SD; *n* = 4) after 3-min incubation in plasma; final volume 160 μL. Exposure to the tested compounds even at the concentrations of 0.5 μmol/mL and above was shown to significantly influence the value of APTT. Statistically significant changes are marked with red frame (*p* < 0.05)

Regarding the effects of gadolinium complexes on the APTT (Fig. 3), there were no statistically significant changes only at the lowest concentration tested—0.1 μmol/mL. The conducted studies showed that all tested compounds in the concentration range of 0.5–1.5 μmol/mL statistically significantly prolonged the APTT value. The statistically significant differences exceed the range of reference values for APTT, which is 26.7–40.0 s.

**Fig. 4:**
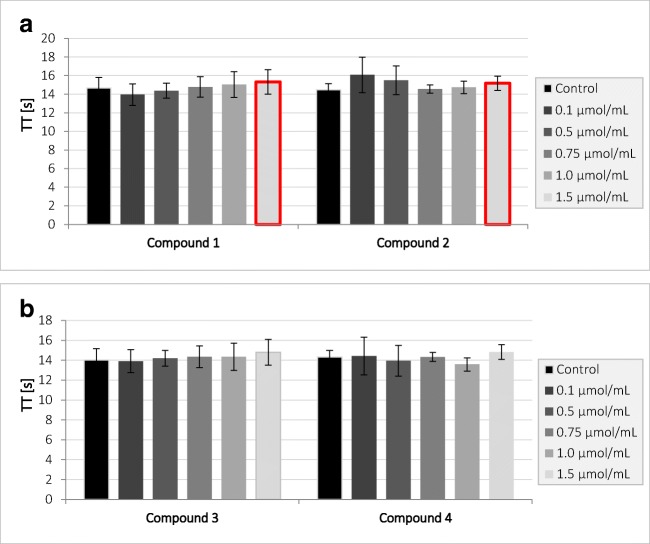
The effects of gadolinium complexes **a** compounds **1** and **2**; **b** compounds **3** and **4** on thrombin time (TT) (mean ± SD; *n* = 4) after 2-min incubation in plasma; final volume 210 μL. All tested compounds apart from the highest concentration of compounds **1** and **2** did not affect TT. Compounds **1** and **2** at 1.5 μmol/mL significantly prolonged TT. Statistically significant changes are marked with red frame (*p* < 0.05)

It was shown that compounds **1** and **2** at concentration of 1.5 μmol/mL statistically significantly affect TT value (Fig. 4) and contribute to its increase; however, these differences are within the reference range for TT (14.0–18.0 s). Compounds **1** and **2** in the remaining concentrations, as well as compounds **3** and **4**, showed no statistically significant changes for TT, which suggests that they do not affect the process of fibrin polymerization.

### RBC Lysis Assay

**Fig. 5:**
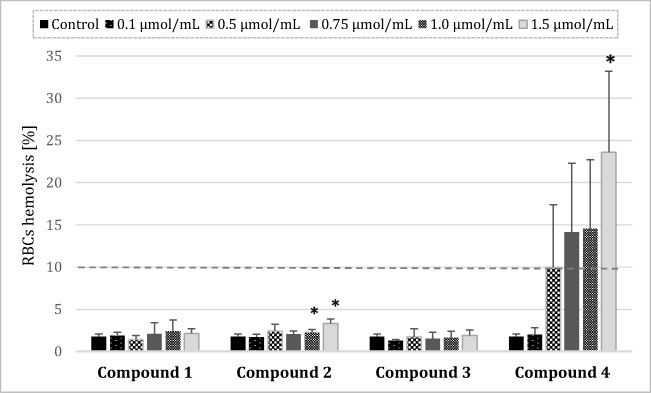
Percentage of hemolysis obtained from the interaction of gadolium complexes with iminodiacetic acid derivatives with 2% RBC (red blood cell) suspension, compared to the positive control Triton X-100 at 0.2% (100% hemolysis) (mean ± SD; *n* = 4), *p < 0.05 vs. control. A statistically significant increase in the rate of hemolysis was documented for the highest concentrations of compound **2**; however, the value of hemolysis did not exceed 5%. Therefore, it might be concluded that compounds **1**–**3** do not show adverse effects on the integrity of RBCs membrane. Compound **4** even at the concentration of 0.5 μmol/mL contributed to the erythrocyte hemolysis exceeding 10%, which is regarded as clinically important

In studies on the effects on the integrity of the erythrocyte membrane, statistically significant results in comparison with saline control were obtained for compound **2** at concentrations of 1.0–1.5 μmol/mL (Fig. 5), which indicates that compound in the highest concentrations caused red blood cell breakdown. However, the values of hemolysis did not exceed 5%; therefore, we assume that compound **2** is biocompatible. In the case of compound **4**, the percentage of hemolysis was higher than 10% at the concentration of 0.5 μmol/mL and above, which gives evidence about disintegration of erythrocyte membrane.

### RBCs Morphology

**Fig. 6:**
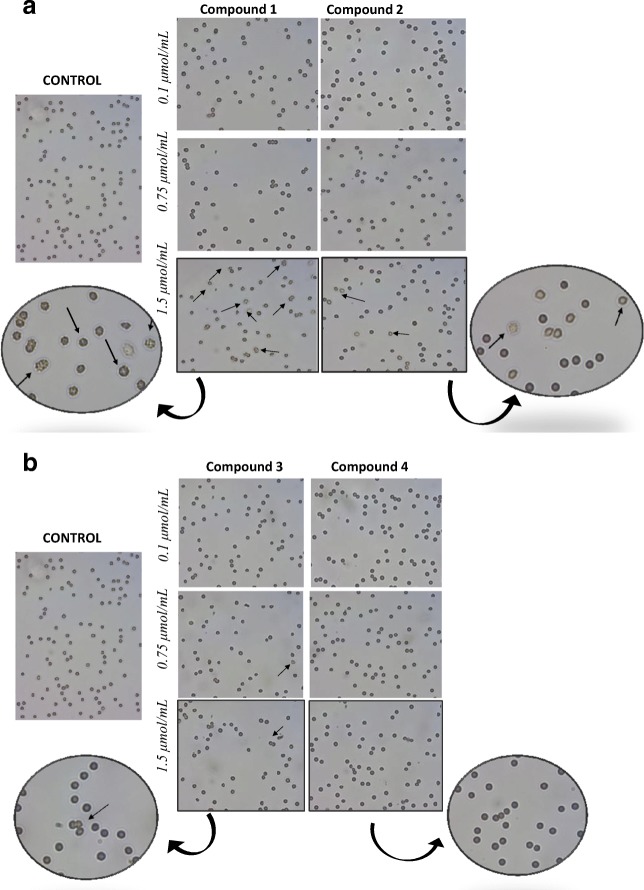
**a** Effect of gadolinium complexes (**1**, **2**) with iminodiacetic acid derivatives on erythrocyte morphology. Two percent erythrocyte suspension was treated at 37 °C for 60 min with indicated concentrations (0.1–1.5 μmol/mL) of compounds **1**–**2**. Representative phase-contrast images are shown (magnification of 400 times). Compounds **1** and **2** at the highest concentration tested contributed to the formation of echinocytes (marked with arrows). **b** Effect of gadolinium complexes (**3**, **4**) with iminodiacetic acid derivatives on erythrocytes morphology. Two percent erythrocyte suspension was treated at 37 °C for 60 min with indicated concentrations (0.1–1.5 μmol/mL) of compounds **3**–**4**. Representative phase-contrast images are shown (magnification of 400 times). Single echinocytes were recognized in the case of compound **3**

During the microscopic evaluation of the erythrocyte morphology, the tendency to form echinocytes was observed at the highest concentrations (1.5 μmol/mL) of compounds **1** and **2** (Fig. 6). Single echinocytes were also distinguished in the case of samples incubated with compound **3** at concentrations of 0.75 and 1.5 μmol/mL. Compound **4** had no effect on the shape of red blood cells at any concentration tested.

### Viability and Integrity of HUVEC Cells

For the measurements of viability of endothelial cells, we applied a novel cell-based method which enabled permanent monitoring of the condition and integrity of cell monolayer—real-time cell electric impedance sensing system xCELLigence (RTCA-DP).

**Fig. 7:**
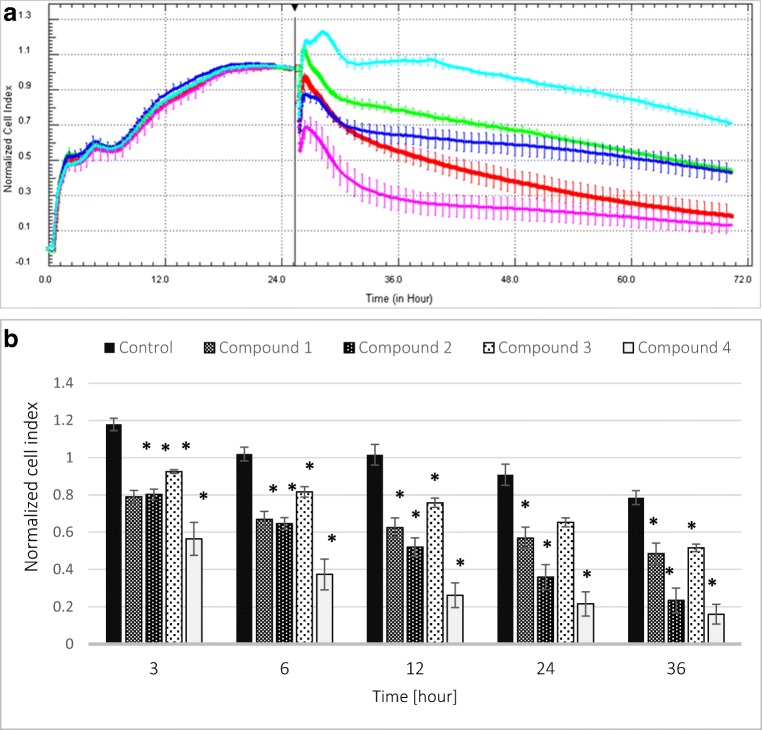
**a** The influence of gadolinium complexes (**1**–**4**) at the concentration of 1 μmol/mL on the viability and integrity of HUVEC cells expressed as Normalized Cell Index. Light blue–control, dark blue–compound **1**, red–compound **2**, green–compound **3**, pink–compound **4**. **b** The effects of gadolinium complexes on the value of Normalized Cell Index at selected time points. All tested complexes in every time point significantly decreased the value of nCI; *p < 0.05

During the 48-h analysis, all tested compounds at a concentration of 1 μmol/mL significantly decreased Cell Index (CI) value (Fig. 7), which gives evidence of reduced integrity of vascular endothelial cells. Two-way ANOVA analysis revealed that the greatest impact on cell survival exhibited compound **4**. In the case of this compound, the reduction of cell integrity and their viability was recorded as the most profound.

### Morphology of HUVEC Cells

**Fig. 8:**
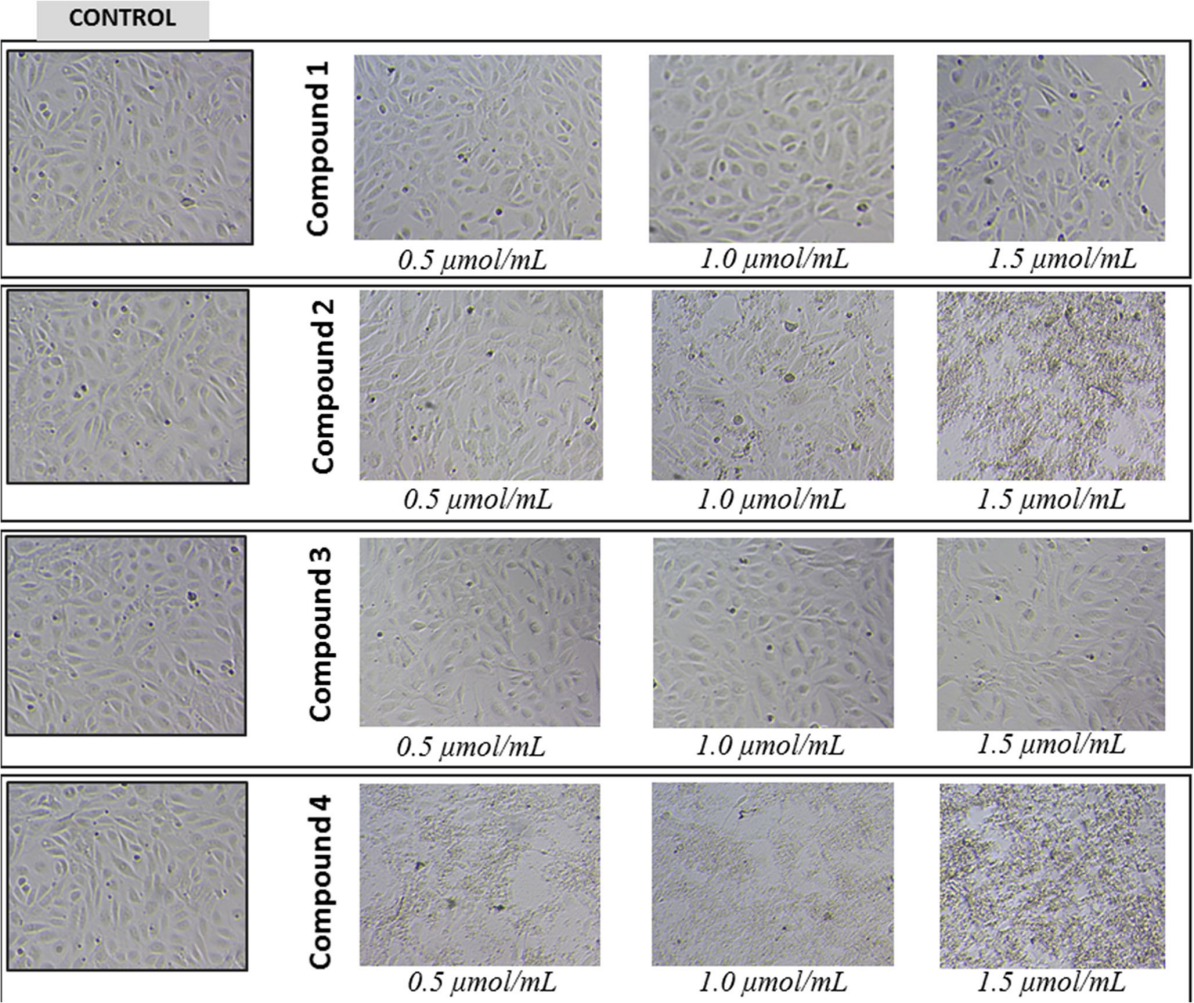
The effect of gadolinium complexes with iminodiacetic acid derivatives on morphology of HUVECs. HUVECs cultured in monolayer on 48-well plates were stimulated with indicated concentrations (0.5–1.5 μmol/mL) of compounds **1**–**4**. Representative phase-contrast images are shown (magnification of 100 times). Compounds **1** and **3** over the entire concentration range did not contribute to the changes in cell morphology. Compound **2** at 1 μmol/mL caused cell membrane disintegration, while compound **4** even at the lowest concentration contributed to the lysis of cells

The effects of gadolinium complexes on the HUVEC cells were monitored for 24 h. Compounds **1** and **3** over the entire concentration range did not contribute to the changes in cell morphology (Fig. 8). Compound **2** at 1.0 and 1.5 μmol/mL caused cell membrane disintegration, while compound **4** even at the lowest concentration contributed to the lysis of cells.

## Discussion

The chemical bonds in gadolinium-based contrast agents (CAs) are made of a gadolinium ion and a carrier molecule which is called a chelating agent. This moiety modifies the distribution of gadolinium within the body to overcome its toxicity while maintaining its contrast properties. Gadolinium-based CAs are used intravenously to enhance the quality of diagnostic MR images [23].

The primary physiological response to any chemical substance, including CAs administered intravenously, involves the interaction with blood components or endothelial cells and usually is regarded as unwanted activity. Therefore, within this study, we assessed the effect of four gadolinium-based potential CAs on integrity of erythrocyte membrane and basic hemostasis parameters by measuring PT, APTT, and TT. The next step of the study was to evaluate the influence of gadolinium complexes on the viability of human endothelial cells. All blood compatibility experiments reported in this manuscript were done by addition of gadolinium complexes dissolved in water to plasma or erythrocyte suspension and the results were compared with the effects of the identical volumes of water to offset any dilution effects.

The hemocompatibility of a novel compound might be evaluated using the blood coagulation assay [3]. Blood coagulation is defined as a complex cascade process whereby soluble plasma proteins become activated in response to vascular injury, leading to the formation of a fibrin clot [4]. The activated partial thromboplastin time (APTT), which shows the activity of coagulation factor XII, XI, IX, can be used to investigate the effect of novel compounds on the intrinsic coagulation pathway. In turn, prothrombin time (PT) specifically evaluates the presence of factors VII, V, and X, prothrombin, and fibrinogen and is used to monitor extrinsic coagulation pathway [4].

Within this study, it was found that only compound **2** at concentration of 0.75 μmol/mL and above significantly prolonged prothrombin time; however, it should be noticed that the results were enclosed within the reference values (9.7–14.6 s). It might be concluded that tested gadolinium complexes are biocompatible regarding PT. In the case of the next coagulation parameter, APTT, it was found that all examined complexes contributed to the significant prolongation of its value. We suppose that this phenomenon might result from higher affinity of iminodiacetic acid derivatives towards calcium ions than gadolinium. This, unfortunately, might be associated with higher risk of gadolinium ion release and their toxicity. In our previous paper [14], we evaluated the effects of all respective ligands (iminodiacetic acid derivatives) for gadolinium complexation using CL test (clot formation and fibrinolysis test). The assay is based on the evaluation of the global assay of coagulation and fibrinolysis by continuous measurements of the optical transmittance alterations and enabled to estimate the kinetic parameters of clot formation and lysis. It was reported that all iminodiacetic acid derivatives caused a significant increase in the thrombin time (↑Tt) when tested at the highest concentrations (4.0 μmol/mL). Furthermore, it was found that the ligands caused a significant increase in the plasma clotting time (↑Tf) and concomitant decrease in the initial plasma clotting velocity (↓Fvo). These results were caused by the inhibitory properties of the compounds towards enzymatic activity of thrombin [14]. Within the current paper, we reported that gadolinium complexes apart from the highest concentration of compounds **1** and **2** did not affect the process of fibrin polymerization (constant TT), which might suggest that the reaction of gadolinium complexation alters the profile of interaction with thrombin and does not contribute to the inhibition of amidolytic activity of the enzyme.

When the drug molecule is administered intravenously the interaction between a xenobiotic and plasma proteins, a series of interactions with blood cells might also be initiated. Within this scientific area, biocompatibility refers to the quantification of cellular and plasma components of the blood. Generally, the effects on erythrocytes are evaluated through hemolysis test, which is considered a simple and reliable measurement for estimating blood biocompatibility [4]. The effect of an intravenously administered drug on RBCs is a crucial issue, since enhanced hemolysis can be detrimental for the human organism. Within this study, we found that complexes **1**–**3** did not lead to the RBC hemolysis exceeding 5%, while complex **4** contributed to approximately 20–25% hemolysis. This observation is of vital importance when referring to a previous study which demonstrated that a hemolysis should be considered cytotoxic when its rate exceeds 10% of that observed in the positive control (0.2% Triton X-100) [24]. Thus, it can be assumed that complexes **1**–**3** are not toxic towards RBCs over the entire concentration range. When comparing the hemolysis results obtained for complex **4** with properties of N-(3-bromo-2,4,6-trimethylacetanilide)-iminodiacetic acid, which is a ligand for gadolinium [14], we can assume that the reaction of complexation does not affect the properties of compound to interact with erythrocyte membrane since the percentage of hemolysis was within the same range.

Healthy erythrocytes usually have the shape of a biconcave disk or discocyte. This shape allows gas exchange and guarantees greater deformability of the erythrocyte required for passing through the capillaries. There are also other types of shapes which are considered physiological: echinocytes and stomatocytes. The erythrocyte maintains the shape of the discus in the pH range of 6.3–7.9. Above pH 7.9, the blood cells undergo echinocyte-echinosferocyte transformation, and erythrocytes adopt the shape of the spherocyte. Further increase in pH causes hemolysis of erythrocytes [25]. During the microscopic evaluation of the erythrocyte morphology, a tendency to form echinocytes was noticed for compounds **1** and **2**. Under the microscope, the echinocytes can be recognized by regularly distributed, numerous, acute, or bluntly terminated cytoplasmic appendages [26]. We presume that formation of echinocytes is related to changes in the organization of cell membrane components after co-treatment with gadolinium complexes.

The vascular endothelium is an important autocrine and paracrine organ that participates not only in regulation of vascular wall functions [27] but also maintains blood fluidity and prevents formation of thrombus [28]. Endothelial monolayer is also engaged in each step of the process of clot formation and fibrinolysis since it takes part in platelet activation and produce factors involved in the coagulation cascade and fibrinolytic system [29]. Negative effects and consequent damage to barrier functions of the vascular endothelium are a commonly known cause of serious disturbances of local homeostasis, causing inflammation, and various pathologies [30]. To the best of our knowledge, this is the first study which evaluated the effect of gadolinium-based CAs on the barrier properties of endothelium. A real-time cell electric impedance sensing system was used to monitor dynamic changes in HUVEC impedance evoked by gadolinium complexes (Fig. 7a, b). After 12 h of cell culture, the nCI of HUVECs treated with gadolinium complexes **1**–**4** (1.0 μmol/mL) was significantly lower than the nCI of unstimulated cells. Similarly, after 24 and 36 h, the results obtained in our study show that the impedance of HUVECs upon the stimulation with gadolinium complexes was lower than in control cells which indicates inhibition of the growth of cells. It can also be implied that the lower impedance of HUVECs upon the treatment with gadolinium complexes may occur due to the decrease in integrity of the monolayer. These results are supported by the microscopic evaluation of HUVECs morphology. As presented in Fig. 8, compound **4** even at the lowest concentration tested contributed to the damage of membrane leading to the cell lysis. Microscopic examinations proved that compound **4** caused major changes in morphology of HUVEC cells and led to the complete destruction of endothelium. Considering the wide profile of endothelium cells’ functions, we can expect many side effects after intravenous administration of this gadolinium complex.

## Conclusions

The current article presents the in vitro biocompatibility study of four gadolinium complexes with derivatives of iminodiacetic acid. The studies of prothrombin time showed that tested compounds do not affect significantly its value. The measurements of activated partial thromboplastin time revealed significant prolongation in the presence of all tested gadolinium complexes which might give evidence of higher affinity of iminodiacetic acid derivatives towards calcium ions than gadolinium. The results of thrombin time showed that the compounds do not affect the process of fibrin polymerization. On the basis of the hemolysis assay, we conclude that compounds **1**–**3** do not exert unfavorable effect on the integrity of erythrocyte membrane over the entire concentration range and do not contribute to hemolysis. However, the studies on HUVECs using real-time cell electric impedance sensing system showed that all gadolinium complexes contribute to the decrease in HUVEC impedance, which corresponds to diminished viability and integrity of cells. To sum up, the study describes biocompatibility of gadolinium-based contrast agent using various in vitro methods. The most favorable safety profile presents complexes **1**, **2**, and **3**; however, it should be emphasized that further in vitro experiments should be conducted in order to determine the safety profile of gadolinium complexes with iminodiacetic acid derivatives. Furthermore, another important issue that still remains a challenge is in vitro to in vivo correlation of toxicity data.

## Electronic supplementary material

ESM 1 (DOCX 56 kb)
