# Supplementary material for: Biocompatibility Studies of Gadolinium Complexes with Iminodiacetic Acid Derivatives
Source: Biol Trace Elem Res. 2018 Sep 13;189(2):426–36. doi: 10.1007/s12011-018-1496-6 (PMC6469645; doi:10.1007/s12011-018-1496-6)
Supplement: Supplementary file 1 — (DOCX 56 kb) [file 12011_2018_1496_MOESM1_ESM.docx]

**BIOCOMPATIBILITY STUDIES OF GADOLINIUM COMPLEXES WITH IMINODIACETIC ACID DERIVATIVES**

**Magdalena Markowicz-Piasecka^1*^, Agata Skupień^2^, Elżbieta Mikiciuk-Olasik^3^, Joanna Sikora^1^**

^1^ Laboratory of Bioanalysis, Department of Pharmaceutical Chemistry, Drug Analysis and Radiopharmacy, Medical University of Lodz, ul. Muszyńskiego1, 90-151 Lodz, Poland; e-mail: magdalena.markowicz@umed.lodz.pl, joanna.sikora@umed.lodz.pl

^2^ Students Research Group, Laboratory of Bioanalysis, Department of Pharmaceutical Chemistry, Drug Analysis and Radiopharmacy, Medical University of Lodz, ul. Muszyńskiego 1, 90-151 Lodz, Poland; agata.sk123@gmail.com

^3^ Department of Pharmaceutical Chemistry, Drug Analysis and Radiopharmacy, Medical University of Lodz, ul. Muszyńskiego 1, 90-151 Lodz, Poland; e-mail: elzbieta.mikiciuk-olasik@umed.lodz.pl

**Figure S1.** The effects of gadolinium complexes **1** - **4** on fibrinogen concentration (mean ± SD; *n* = 5) after 3 min incubation in plasma; final volume 160 μL. None of the tested compounds significantly affected fibrinogen level.
